# Ethical and Psychosocial Implications of Genomic Newborn Screening

**DOI:** 10.3390/ijns7010002

**Published:** 2021-01-09

**Authors:** Harvey L. Levy

**Affiliations:** 1 Division of Genetics and Genomics, Boston Children’s Hospital, Boston, MA 02115, USA; Harvey.Levy@childrens.harvard.edu; 2 Department of Pediatrics, Harvard Medical School, Boston, MA 02115, USA

**Keywords:** genomic sequencing, ethics, interpretation, newborn screening

## Abstract

The potential for genomic screening of the newborn, specifically adding genomic screening to current newborn screening (NBS), raises very significant ethical issues. Regardless of whether NBS of this type would include entire genomes or only the coding region of the genome (exome screening) or even sequencing specific genes, the ethical issues raised would be enormous. These issues include the limitations of bioinformatic interpretation of identified variants in terms of pathogenicity and accurate prognosis, the potential for substantial uncertainty about appropriate diagnosis, therapy, and follow-up, the possibility of much anxiety among providers and parents, the potential for unnecessary treatment and “medicalizing” normal children, the possibility of adding large medical costs for otherwise unnecessary follow-up and testing, the potential for negatively impacting medical and life insurance, and the almost impossible task of obtaining truly-informed consent. Moreover, the potentially-negative consequences of adding genomic sequencing to NBS might jeopardize all of NBS which has been and continues to be so beneficial for thousands of children and their families throughout the world.

## 1. Evolution of Newborn Screening

Dr. Robert Guthrie, the founder of newborn screening (NBS), and I were sharing a taxi in New York in the late 1980’s or early 1990’s heading for the airport when again he brought up the subject of NBS for histidinemia. He had modified his famous bacterial inhibition assay, the one he had developed for phenylalanine to identify phenylketonuria (PKU), so that maple syrup urine disease (MSUD) and homocystinuria (HCU) could be added to NBS. This latest modification semi-quantitatively measured histidine so that now histidinemia could be added to NBS. But I knew histidinemia very well. In the Massachusetts NBS Program we had identified many cases through urine screening [1] and in my follow-up of these children I knew that histidinemia was benign [2], a disorder that Archibald Garrod would have defined as a “metabolic sport” [3]. So I argued that histidinemia should not be added to NBS, that screening for it would be of no benefit and that indeed, it would result in much anxiety to the parents and clinicians, would “medicalize” the children by falsely labeling then as having a disease, might result in their being unnecessarily put on a difficult and expensive diet and therefore, it would be unethical to screen for histidinemia. Guthrie was a brilliant medical researcher but he was not a clinician and had never seen a child with histidinemia. Nevertheless, he was so dedicated to preventing intellectual disability that he believed every case report of disability, however misleading, and rejected every study to the contrary. NBS at that time was only possible for disorders that led to disabilities, such as PKU, MSUD, HCU, congenital hypothyroidism, congenital adrenal hyperplasia, and sickle cell disease so ethics and psychosocial issues had not yet become major considerations. 

A few years later, however, tandem mass spectrometry (MS-MS), which greatly expanded NBS, came into being. With this technology a single assay could screen for many metabolic disorders rather than each disorder requiring its own assay. It soon became clear that although this technology added many important disorders to NBS and prevented substantial disability as well as sudden death, expanded NBS (ENBS) also resulted in identifying benign disorders and benign mild variants of otherwise disabling disorders. In fact, it is likely that the majority of infants identified by MS-MS have a benign finding [4,5,6]. This raised issues such as what to report to attending physicians, which findings should be called to the attention of parents, which findings required confirmatory assessment and perhaps treatment, and how parents received and perceived this information. Thus, ethical and psychosocial concerns became major considerations in NBS [7,8]. 

Robert Guthrie was a genius. Like so many geniuses he came to an idea and then devoted his life to making this idea a reality that changed the world. His development of newborn screening required two components—a test which was the bacterial inhibition assay that semi-quantitatively measured phenylalanine and a blood specimen that could easily and safely be obtained from the newborn infant and readily transported to a central laboratory [9]. Of these two components, Guthrie maintained that the one he would be best remembered for was the filter paper blood specimen, for he correctly recognized that the bacterial assay would be replaced but the dried blood specimen would remain and allow for many additional tests that would benefit babies and their families [10]. Indeed, that has been true. From its beginning as a single screening for PKU, additional tests were added in stages. First, in the mid- and late-1960’s bacterial assays that would identify galactosemia, MSUD, and HCU, then, in the mid 1970’s, a test for congenital hypothyroidism was added, followed by tests for sickle cell disease and congenital adrenal hyperplasia in the late 1970’s, biotinidase deficiency in the 1980’s and early 1990’s, cystic fibrosis and then tandem mass spectrometry in the late 1990’s and early 2000’s, and, most recently, NBS for severe combined immunodeficiency, Pompe disease, adrenoleukodystrophy, mucopolysaccharidosis type 1, and spinal muscular atrophy. However, even Guthrie could but imagine how valuable the blood “spot” would turn out to be and that it would be used for many things beyond NBS, including genomic sequencing. 

In 1987 McCabe and colleagues set the stage for sequencing by recognizing that since the blood impregnated within the filter paper was whole blood it contained DNA. They then developed a method for extracting the DNA [11]. Subsequently, dried blood in filter paper became used for molecular diagnosis in patients with clinical symptoms [12] and, most recently, a number of studies have examined next-generation sequencing in the dried blood specimens for the possibility that it could be used in NBS [13,14,15,16].

## 2. Potential Applications of Genomics Screening

There are several potential applications of next-generation sequencing in NBS [17,18].

### 2.1. Targeted Genotyping

This application would be to either analyze a panel of known pathogenic gene variants in a single gene or to sequence the entire coding sequence of a selected set of genes. The former is used today in some NBS programs for second-tier screening, i.e., to follow an abnormal initial NBS result for cystic fibrosis, galactosemia, or medium chain acyl-CoA dehydrogenase deficiency in order to better define the initial abnormality. Sequencing an entire coding sequence of selected genes would, of course, potentially identify more infants with genetic disorders than NBS currently identifies but might also result in identifying many more infants with benign disorders as well as infants with variants of undefined significance (VUS), the vast majority of which are simply normal nucleotide variations but for which required costly and lengthy follow-up and continued uncertainty could overwhelm the system. 

### 2.2. Genome-Wide Sequencing

This could be either whole-exome sequencing (WES), i.e., sequencing only the coding sequences (exons) in the genome or whole-genome sequencing (WGS), i.e., sequencing the entire genome, all three billion nucleotides. Both of these possibilities offer the advantage of detecting virtually any genetic disorder but the very significant disadvantage of resulting in even many more VUSs than sequencing only selected genes (see above) and requiring confirmatory evaluations and likely still-inconclusive results with perhaps long periods of follow-up and additional testing in very large numbers of clinically-normal infants. If targeted sequencing could overwhelm the follow-up system, the far more extensive demands for follow-up of genome-wide sequencing could not imaginably be accommodated by the current follow-up system.

## 3. Ethical and Psychosocial Implications

The potential for genomic NBS has raised ethical and psychosocial concerns to a new level. The original NBS included only a very few disorders. ENBS included many more disorders but all of a metabolic nature. Genomic NBS, however, could expand NBS beyond anything originally imagined. Certainly, many more metabolic disorders could be identified but beyond metabolics, genomic NBS could include a huge number of non-metabolic clinical genetic disorders such as developmental defects, cerebral malformations, genetic causes of epilepsy, immunologic disorders, and hematologic disorders. One can imagine the ethical and psychosocial concerns raised by this degree of NBS expansion. Among the questions would be the purpose of identifying so many of these disorders in phenotypically-normal newborn infants, whether the negative effects would outweigh any good from genomic sequencing, whether bioinformatics would allow the precision of interpretation required to identify important findings and exclude insignificant changes, whether interpretation of the genotypic results could predict outcomes with any degree of accuracy, how the information derived from genomic sequencing could be best acted upon, and how the information would be received and perceived by physicians and, especially, by parents. 

Thus it is fitting for the *International Journal of Neonatal Screening* (*IJNS*) to devote this issue to ethical and psychosocial issues of NBS. These issues include the diagnostic and prognostic dilemmas of variant interpretation, how to handle the identification of carriers, identifying benign variants with the inevitable uncertainty and anxiety that this produces, unnecessary treatment, the potential for very large additional medical costs of confirmatory testing and unnecessary treatment, the identification of late-onset disorders with the need for continuous follow-up and uncertainty of early therapy, the need for informed consent and the difficulty of providing the correct information for appropriate consenting, the potential for adverse long-term consequences of insurance for the family as well as the infant, the need for additional geneticists and other specialists to handle, educate, and treat these newly-detected newborns, and the dreaded possibility that standard NBS for PKU, congenital hypothyroidism, and other well-established screening might be threatened [17,18,19]. All of these and many more issues must be carefully examined [20,21], as they will be in this special issue of the *IJNS*.
